# Later Age at Onset of Independent Walking Is Associated With Lower Bone Strength at Fracture‐Prone Sites in Older Men

**DOI:** 10.1002/jbmr.3099

**Published:** 2017-03-27

**Authors:** Alex Ireland, Stella Muthuri, Joern Rittweger, Judith E Adams, Kate A Ward, Diana Kuh, Rachel Cooper

**Affiliations:** ^1^ School of Healthcare ScienceManchester Metropolitan UniversityManchesterUK; ^2^ MRC Unit for Lifelong Health and Ageing at UCLLondonUK; ^3^ Division of Space PhysiologyInstitute of Aerospace MedicineGerman Aerospace CenterCologneGermany; ^4^ Department of Pediatrics and Adolescent MedicineUniversity of CologneCologneGermany; ^5^ Clinical RadiologyManchester Royal InfirmaryManchesterUK; ^6^ Academic Health ScienceManchester University Hospital NHS Foundation TrustManchesterUK; ^7^ Division of InformaticsImaging, and Data SciencesFaculty of Biology, Medicine, and HealthUniversity of ManchesterManchesterUK; ^8^ MRC Lifecourse Epidemiology UnitUniversity of SouthamptonSouthamptonUK; ^9^ MRC Elsie Widdowson LaboratoryCambridgeUK

**Keywords:** BONE MASS, BONE MINERAL DENSITY, MOTOR DEVELOPMENT, GROWTH, AGEING

## Abstract

Later age at onset of independent walking is associated with lower leg bone strength in childhood and adolescence. However, it is unknown whether these associations persist into older age or whether they are evident at axial (central) or upper limb sites. Therefore, we examined walking age obtained at age 2 years and bone outcomes obtained by dual‐energy X‐ray absorptiometry (DXA) and peripheral quantitative computed tomography (pQCT) scans at ages 60 to 64 years in a nationally representative cohort study of British people, the MRC National Survey of Health and Development. It was hypothesized that later walking age would be associated with lower bone strength at all sites. Later independent walking age was associated with lower height‐adjusted hip (standardized regression coefficients with 95% confidence interval [CI] –0.179 [–0.251 to –0.107]), spine (–0.157 [–0.232 to –0.082]), and distal radius (–0.159 [–0.245 to –0.073]) bone mineral content (BMC, indicating bone compressive strength) in men (all *p* < 0.001). Adjustment for covariates partially attenuated these associations, primarily because of lower lean mass and adolescent sporting ability in later walkers. These associations were also evident for a number of hip geometric parameters (including cross‐sectional moment of inertia [CSMI], indicating bone bending/torsional strength) assessed by hip structural analysis (HSA) from DXA scans. Similar height‐adjusted associations were also observed in women for several hip, spine, and upper limb outcomes, although adjustment for fat or lean mass led to complete attenuation for most outcomes, with the exception of femoral shaft CSMI and spine bone area (BA). In conclusion, later independent walking age appears to have a lifelong association with bone strength across multiple skeletal sites in men. These effects may result from direct effects of early life loading on bone growth and mediation by adult body composition. Results suggest that late walking age may represent a novel risk factor for subsequent low bone strength. Existing interventions effective in hastening walking age may have positive effects on bone across life. © 2017 The Authors. *Journal of Bone and Mineral Research* Published by Wiley Periodicals Inc.

## Introduction

The large muscle and impact forces associated with locomotion have a pronounced effect on lower limb bone strength; athletes engaged in sprinting and running have up to 23% higher bone mineral content (BMC, an indicator of bone compressive strength) than sedentary controls,^1^ whereas cessation of ambulation in spinal cord injury patients leads to a loss of 30% of lower limb BMC.^2^ Onset of independent walking at around 12 to 18 months of age represents the first postnatal exposure of the lower limbs to these forces. Previous studies have identified strong associations between later attainment of independent walking and other locomotor activities, and lower tibial BMC and cross‐sectional moment of inertia (CSMI, an indicator of bone bending/torsional strength) in young children,^3^ and tibia/hip BMC and tibia CSMI in adolescents.^4^ These associations were partly mediated by lower lean mass in later walkers, which may be acting as a marker of lower physical activity. In the latter study, the Avon Longitudinal Study of Parents and Children (ALSPAC),^4^ examination of sex differences revealed stronger associations in males than females as in previous studies of physical activity and bone.^5^

Later age at onset of independent walking may therefore represent a novel risk factor for low hip bone strength. Given that development of independent walking can be modified in both healthy children^6^ and in groups prone to delayed motor development,^7^, ^8^ this may offer an opportunity for improving lifelong bone health. However, it is unknown whether associations between age at walking onset and bone strength persist into older age. Hip fractures are rare in children and adolescents^9^ but represent the most common fracture site in older individuals.^10^ Given that hip bone strength is an important risk factor for future fracture,^10^ it is relevant to establish the long‐term potential consequences of later development of walking on bone health in later adulthood. In addition, previous studies investigating associations between walking age and bone have only investigated lower limb bone strength, and associations between early life movement and central or upper limb sites such as the spine or distal radius (where fractures are also common and/or lead to a substantial burden on health services) are unknown.

Therefore, this study investigated associations between age at onset of independent walking and hip, spine, and wrist bone strength (indicated by BMC and/or CSMI) in older men and women in the MRC National Survey of Health and Development (NSHD). This British birth cohort study has prospective data on potential confounding and mediating factors in childhood (birth weight, father's socioeconomic position [SEP] and sporting ability) and adulthood (body size, own SEP, and physical activity). It was hypothesized that later walking age would be associated with lower measures of bone strength at all sites and that associations would be stronger in men and partly mediated by lean mass, as in previous observations in an adolescent cohort (ALSPAC).

## Materials and Methods

The MRC National Survey of Health and Development (NSHD) is a birth cohort study, which includes a nationally representative sample of 2547 men and 2815 women of white European descent born in England, Scotland, and Wales during 1 week in March 1946. The cohort has been followed prospectively across life with outcome data for these analyses drawn from a data collection between 2006 and 2010 when participants were 60 to 64 years old.^11^ Study members still alive and for whom a current address in England, Scotland, or Wales was available (*n *= 2856) were invited for assessment at one of six clinical research facilities (CRFs) or to be visited by a research nurse at home. Of these participants, 2229 (78%) were assessed; 1690 (76%) in a CRF. This participating sample is broadly representative of native‐born British men and women of similar age.^12^ Relevant ethical approval was received, and informed consent was obtained from all participants.

### Musculoskeletal assessment

A total of 1690 participants attended one of the six CRFs, at which height was measured using a stadiometer, and weight was measured with portable electronic scales while participants were lightly clothed and unshod. Of these 1690 participants, 1658 had total body, hip, and spine dual‐energy X‐ray absorptiometry (DXA) scans. At each site, scans were acquired using QDR 4500 Discovery (Hologic Inc., Bedford, MA, USA) scanners, software version APEX version 3.1. Of these, 1350 participants attending one of the five CRFs with an XCT 2000 peripheral quantitative computed tomography (pQCT) scanner (Stratec, Pforzheim, Germany) also had pQCT scans of the non‐dominant radius. Scanning protocols were standardized across all sites by distribution of a detailed training protocol booklet and illustrative CD. Results from scans of the European Spine^13^ (ESP number 04‐220) and Forearm^14^ phantom for DXA and pQCT, respectively, were compared between all sites.^15^ Cross‐calibration was performed for DXA according to the method described by Pearson and colleagues^16^ but was not required for pQCT.

From the total body DXA scans, total body (less head) lean and fat mass were recorded. From spine scans, mean bone mineral content (BMC), bone mineral density (BMD), and bone area (BA) for lumbar vertebrae L_1_ to L_4_ were obtained. From the proximal femur scan, total hip BMC, BA, and BMD were assessed. These femoral scans were further analyzed to obtain geometrical characteristics using the hip structural analysis (HSA) module.^17^ The distribution of bone mineral across 5‐mm‐thick sections of the narrowest point of the femoral neck (FN) and at the femoral shaft (FS) at a point 2 cm distal to the lesser trochanter midpoint were assessed. At each site, estimated measures of cross‐sectional area (CSA), cortical thickness (CT), cortical BMD, and cross‐sectional moment of inertia (CSMI, a composite measure of bone mineral density and shape indicating bone stiffness in bending and torsion) were calculated. Repeat scan precision for DXA BMD measures in adults (*n* = 22) was determined in one CRF and was <1% for all measurements.

To assess whether associations with walking age were confined to weight‐bearing bones, pQCT scans at the distal radius (taken at 4% distal‐proximal ulna length) were examined using v6.00B of the software supplied with the machine using a contour mode C2P1, peeling threshold of 169 mg.mm^−1^ to separate bone from surrounding soft tissue. Total BMC (vBMC.tot, mg.mm^−1^), total bone cross‐sectional area (Ar.tot, mm^2^), and trabecular BMD (vBMD.ct, mg.mm^−3^) were assessed. pQCT 4% repeat scan precision in 20 adults was assessed at one CRF and ranged between 1% and 2%.

### Age at onset of independent walking

During an assessment at age 2 years, study participants’ mothers recalled the age (in months) at which their child first walked unaided.

### Covariates

Factors that may potentially confound or mediate the main associations of interest were selected a priori based on previous findings in the literature. These were current height, lean and fat mass, early life factors (birth weight, father's occupational class, and sporting ability), and adult life factors (own occupational class and leisure time physical activity).

Birth weight was extracted from medical records within a few days of birth, and measurements to the nearest quarter‐pound (113 g) were converted to kilograms. As indicators of socioeconomic position (SEP), father's occupation at child age 4 years (or at age 11 or 15 if missing at age 4) and own occupation at age 53 years (or if not available, the most recent measure in adulthood) were both categorized into six groups (I [professional], II [managerial and technical], IIINM [skilled non‐manual], IIIM [skilled manual], IV [partly skilled], and V [unskilled]) using the Registrar General's Social Classification.^18^ At age 13 years, children were graded as above average, average, or below average according to teacher reports of their sporting ability. This measure is used as a marker of motor skills and coordination evidenced by school‐based physical activity and is predictive of leisure‐time physical activity levels in adulthood.^19^ Participation in leisure‐time physical activity at ages 60 to 64 years was ascertained by asking participants to report whether they had undertaken any sports, vigorous leisure activities, or exercises in their spare time, not including getting to and from work, in the last 4 weeks and if so on how many occasions they had done these activities. This was categorized into three groups: inactive (no participation); moderately active (participated 1 to 4 times); most active (participated ≥5 times).

### Statistical analysis

Data were analyzed using the R statistical environment (version 3.1.2, www.r-project.org), for 1215 participants (627 women) with complete DXA data and all covariates. A series of multiple linear regression models were used to examine associations between age at onset of independent walking with each bone outcome, adjusted for each set of covariates. The main models were all sex‐stratified because of previous findings^4^ and evidence of sex interactions when these were formally tested. Model 1 examined the unadjusted associations between walking age and each outcome. Model 2 was adjusted for height only. Model 3 was further adjusted for early life factors (birthweight + father's occupational class + sports ability), whereas further adjustment for adult life factors (adult occupational class + exercise) was performed in model 4. Remaining models considered body composition; models 5 and 6 were further adjusted for fat and lean mass, respectively. Fully adjusted model 7 considered all covariates, ie, lean and fat mass were both entered.

Associations between walking age and bone outcomes are reported as standardized coefficients (indicating the SD change in the outcome associated with one SD change in age at walking). Formal tests of deviation from linearity (ie, inclusion of quadratic terms and modeling age at walking with individuals divided into sixths) have also been performed, but there was no evidence of non‐linearity.

### Sensitivity analyses

To investigate whether associations between walking age and bone outcomes were mediated by differences in pubertal timing, model 7 was further adjusted for pubertal status in the subsample of 586 women and 540 men for whom these data were available. Pubertal status was assessed by a school doctor, through a medical examination and interview.^20^ For women, age at menarche was obtained from mothers’ reports during the examination or retrospectively at age 48 years by self‐report for those women who had not reached menarche at time of assessment. Development in boys was graded by assessment of voice breaking, visible pubic hair and axillary hair, and genital development.^20^ As an alternative approach to assessing independent effects of fat and lean mass, models were also constructed using residual values of fat adjusted for lean and vice versa. The effects of local rather than total body mass were assessed in further models adjusted for lower or upper limb lean and fat mass derived from DXA scans for hip and radius bone outcomes, respectively.

In addition to assessments adjusted using self‐reports of leisure‐time physical activity, models 4 to 7 were alternatively adjusted using accelerometry data in 511 women and 486 men for whom these data were available. Accelerometry data were obtained using a chest‐worn Actiheart movement monitor as reported previously.^21^, ^22^ Briefly, participants wore a monitor for up to 5 consecutive days at ages 60 to 64 years, during which time acceleration and heart rate was measured in 30‐second epochs. Data from each epoch were converted and categorized to intensities relative to 1 standard metabolic equivalent (MET) as follows: sedentary (≤1.5 METs), light (1.5 to 3 METs), and MVPA (>3 METs). Data were adjusted for wear time and diurnal information bias to give an estimate of the time spent in an average day at each level of intensity. Models were adjusted for each band individually and additionally for all three bands simultaneously.

## Results

### Cohort characteristics

Characteristics of the 627 women and 588 men with complete data included in analyses are presented in Tables 1 and 2 There was no sex difference in walking age (*p* = 0.38), but later age at onset of walking was associated with higher child and adult SEP, greater adult height, lower weight and lean and fat mass, and below average sporting ability in both sexes (Supplemental Table S1).

**Table 1:** Characteristics of the MRC National Survey of Health and Development Stratified by Sex (Sample Restricted to Those With Complete DXA Data)

		Men (n = 588)		Women (n = 627)	
Variable		Mean	SD	Mean	SD
Age (years)		63.1	1.2	63.2	1.1
Birthweight (kg)		3.46	0.58	3.39	0.65
Height (m)		1.75	0.06	1.62	0.06
Weight (kg)		84.9	12.8	72.3	13.7
Lean mass (kg)		53.6	6.9	37.2	5.4
Fat mass (kg)		23.7	7.2	29.1	9.2
Walking age (months)		13.7	2.4	13.6	2.3
		*n*	%	*n*	%
Father's occupational class (age 4 years)	I	42	7.1	47	7.5
	II	132	22.4	140	22.3
	IIINM	117	19.9	123	19.6
	IIIM	176	29.9	173	27.6
	IV	89	15.1	116	18.5
	V	32	5.4	28	4.5
Sports ability (age 13 years)	Above average	114	19.4	120	19.1
	Average	367	62.4	445	71.0
	Below average	107	18.2	62	9.9
Own occupational class (age 53 years)	I	75	12.8	14	2.2
	II	275	46.8	268	42.7
	IIINM	69	11.7	220	35.1
	IIIM	126	21.4	38	6.1
	IV	36	6.1	65	10.4
	V	7	1.2	22	3.5
Leisure‐time physical activity (aged 60 to 64 years)	Inactive	355	60.4	354	56.5
	Moderately active	86	14.6	107	17.1
	Most active	147	25.0	166	26.5

Occupational classes: I = professional; II = managerial and technical; IIINM = skilled non‐manual; IIIM = skilled manual; IV = partly skilled; V = unskilled.

**Table 2:** Bone Outcomes From DXA and pQCT Scans at Ages 60 to 64 Years in the MRC National Survey of Health and Development, Stratified by Sex

DXA					
		Men (*n* = 588)		Women (*n* = 627)	
Site	Variable	Mean	SD	Mean	SD
Total hip	BMD (g.cm^‐2^)	1	0.14	0.87	0.13
	BMC (g)	46.8	8	31.4	5.3
	BA (cm^2^)	46.3	4.7	35.3	3.3
Femoral neck	BMD (g.cm^‐3^)	0.99	0.15	0.92	0.15
	CT (mm)	0.19	0.03	0.18	0.03
	CSA (mm^2^)	3.55	0.54	2.87	0.45
	CSMI (mm^4^)	4.39	1.03	2.63	0.64
Femoral shaft	BMD (g.cm^‐3^)	1.75	0.22	1.49	0.2
	CT (mm)	0.67	0.12	0.56	0.1
	CSA (mm^2^)	5.49	0.76	4.19	0.58
	CSMI (mm^4^)	5.85	1.38	3.64	0.85
Spine	BMD (g.cm^‐2^)	1.05	0.18	0.94	0.16
	BMC (g)	74.6	15.5	56.2	11.5
	BA (cm^2^)	70.2	6.7	58.5	5.6
Upper limb	BMD (g.cm^‐2^)	0.85	0.08	0.69	0.07
	BMC (g)	437	68	278	45
	BA (cm^2^)	515	50	404	41

pQCT					
		Men (*n* = 499)		Women (*n* = 500)	
Site	Variable	Mean	SD	Mean	SD
4% radius	Total BMC (mg.mm‐1)	147	27	95	17
	Total CSA (mm^2^)	382	74	292	53
	Trabecular BMD (mg.mm‐3)	205	42	173	43

BMD = bone mineral density; BMC = bone mineral content; BA = bone area; CT = cortical thickness; CSA = cross‐sectional area; CSMI = cross‐sectional moment of inertia.

### Total hip bone outcomes

**Figure 1:**
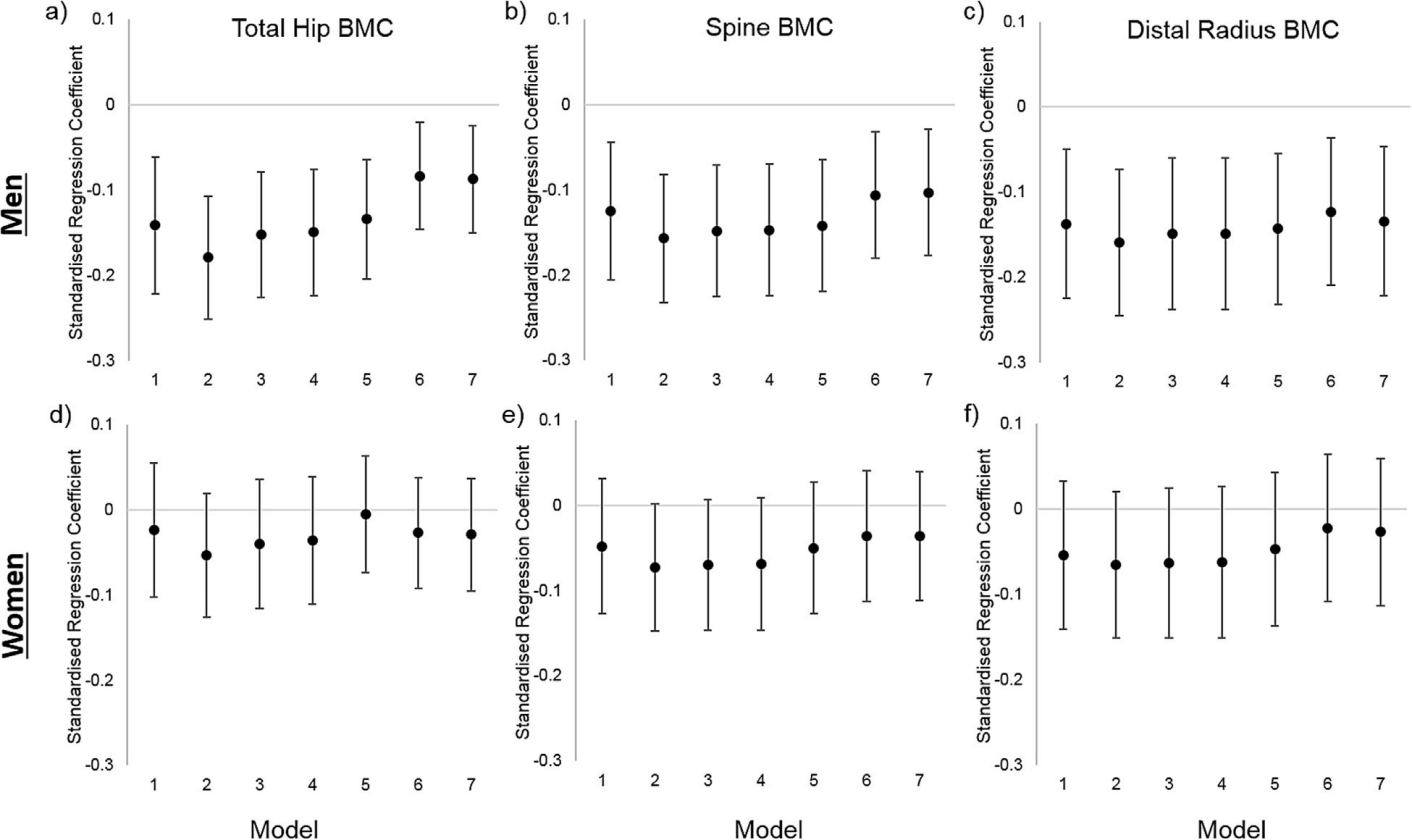
Associations between age at onset of independent walking and total hip, spine and distal radius bone mineral content (BMC) for men and women in the MRC National Survey of Health and Development. Associations are presented as standardised regression coefficients, representing the SD difference in each bone outcome per 1SD increase in age at walking.Footnote: Model 1 predictors: Walking Age, Model 2: Model 1 + Height, Model 3: Model 2 + Birthweight + Father's Occupational Class + Sports Ability, Model 4: Model 3 + Adult Occupational Class + Exercise, Model 5: Model 4 + Fat Mass, Model 6: Model 4 + Lean Mass, Model 7: Model 6 + Fat Mass + Lean Mass.

Unadjusted regression models showed that later walking age was associated with lower total hip BMC in men (Fig. 1 *A*, model 1); similar associations were observed for total hip BMD and BA (Supplemental Table S2). Adjustment for height (model 2) increased the strength of these associations for all variables. Further adjustment for early life factors partially attenuated this association, and further substantial attenuation occurred after adjustment for lean but not fat mass for all three outcomes (although only for BMD was this attenuation complete). There was no association between walking age and hip bone outcomes in any model in women (all *p* > 0.1).

### Lumbar spine bone outcomes

In men, later walking age was associated with lower spine BMC (Fig. 1 *B*, model 1), BMD, and BA values (Supplemental Table S2) in unadjusted models and, as with hip bone outcomes, strength of associations increased after adjustment for height in model 2 for all outcomes. Adjusting for early life factors and current fat mass had little effect on these estimates, whereas further adjustment for lean mass led to full attenuation of BMD and partial attenuation of BMC/BA associations (model 6).

Although no associations between walking age and spine outcomes were observed in women in the unadjusted model, weak associations between later walking and lower spine BMC and BA were found after adjustment for height. These associations were attenuated by adjustment for lean and/or fat mass for BMC but not BA.

### Hip geometry outcomes

**Figure 2:**
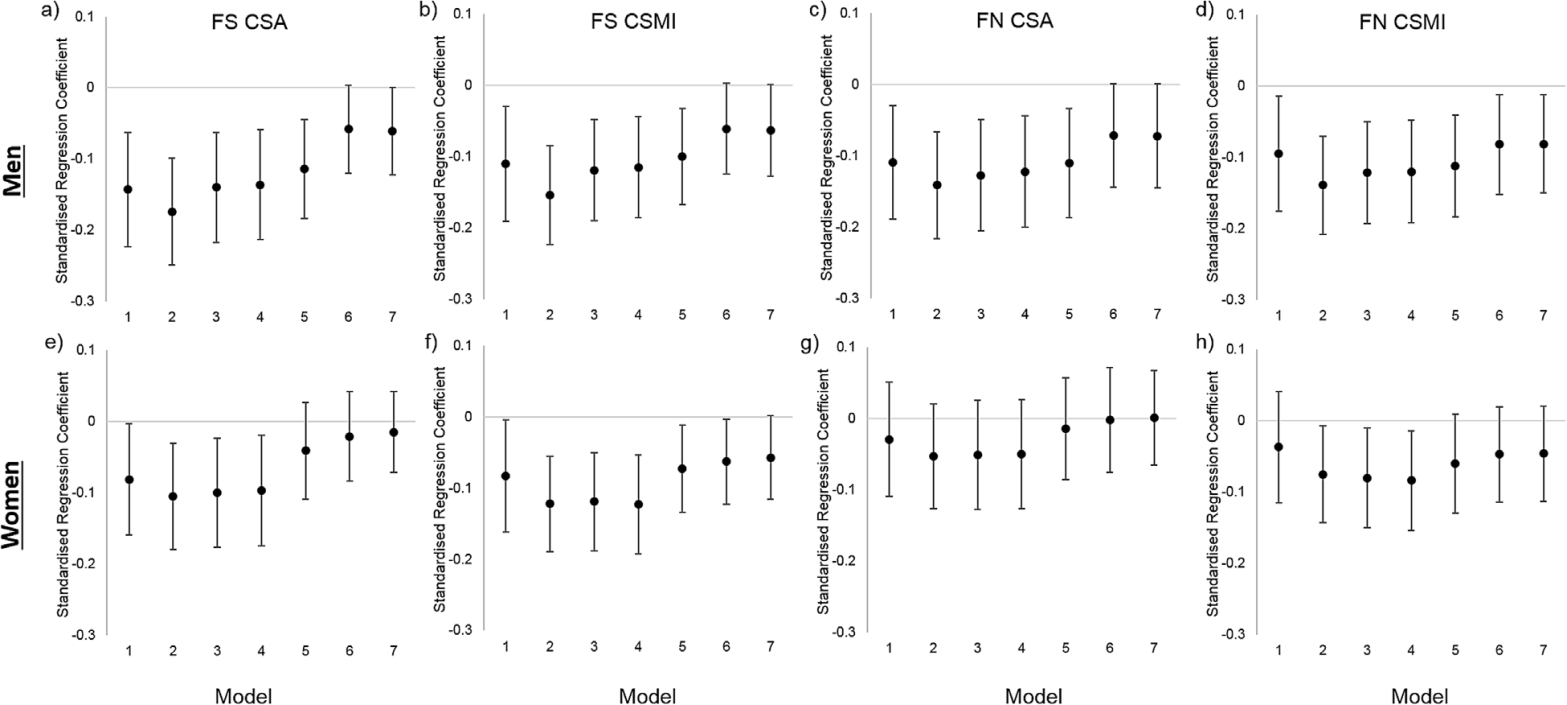
Associations between age at onset of independent walking and femoral shaft (FS) and femoral neck (FN) hip structural analysis (HSA) outcomes for men and women in the MRC National Survey of Health and Development. Associations are presented as standardised regression coefficients, representing the SD difference in each bone outcome per 1SD increase in age at walking. CSA—cross‐sectional area, CSMI—crosssectional moment of inertia.Footnote: Model 1 predictors: Walking Age, Model 2: Model 1 + Height, Model 3: Model 2 + Birthweight + Father's occupational Class + Sports Ability, Model 4: Model 3 + Adult Occupational Class + Exercise, Model 5: Model 4 + Fat Mass, Model 6: Model 4 + Lean Mass, Model 7: Model 6 + Fat Mass + Lean Mass.

For both the femoral shaft and femoral neck sites, later walking age was associated with lower values in all outcomes (CSA, CT, BMD, and CSMI) in men with little attenuation after adjustment for height, early life factors, or adult life factors (Fig. 2 *A–D* and Supplemental Table S3, models 1 to 5). Adjustment for body composition, particularly lean mass, partially (or completely for BMD and pCT) attenuated these associations.

In women, there was no association with BMD or CT in any model at either site (Supplemental Table S3, all *p* > 0.15). Later walking age was associated with lower CSA and CSMI (Fig. 2 *E–H*); these associations were partially (CSMI) or wholly (CSA) attenuated by adjustment for either fat or lean mass.

### Distal radius bone outcomes

Distal radius pQCT outcomes were also examined in the subcohort of 499 men and 500 women from whom these scans had been obtained. Later walking age was associated with lower total BMC (Fig. 1 *C*) and was weakly associated with lower total CSA (Supplemental Table S4) in men; these associations were little affected by adjustment in models 2 to 7. There was no association between walking age and pQCT outcomes in women.

### Sensitivity analysis

Adjustment for pubertal status did not result in substantial attenuation of associations for any bone outcome. Use of upper limb or lower limb lean and fat mass rather than whole body measures or adjustment by lean and fat mass residuals rather than raw values did not substantially affect associations. Similarly, use of accelerometry rather than self‐reported physical activity data did not substantially influence results. Although results are shown only for participants with complete data, analyses run using maximum available samples produced similar findings (not reported).

## Discussion

In a large, national cohort study, later attainment of independent walking was associated with lower hip bone strength (indicated by BMC and CSMI) in men aged 60 to 64 years. These associations were also evident at central (L_1_ to L_4_ spine) and upper limb sites, and partly explained by differences in lean mass. Similar associations were also observed for several hip, spine, and upper limb variables in women after adjustment for height, although with the exception of femoral shaft CSMI and spine BA, associations were completely attenuated by adjustment for fat or lean mass. These results are in agreement with the hypothesis that later walking age would be associated with lower bone strength at all sites, with stronger associations in men.

### Comparison with previous findings

That we found later walkers had lower bone strength at ages 60 to 64 years is similar to previous findings in young children^3^ and adolescents.^4^ The current study extends previous work by examining an older cohort, results suggesting that these associations persist into older age when low bone strength and fractures become more prevalent. As reported in a previous study,^4^ we also found that associations between walking age and bone strength were more pronounced in men than in women, although lower hip bending strength (CSMI) was observed in later walkers of both sexes. Previous studies had focused on associations between walking age and lower limb bone strength indicators, on the basis that locomotory competence would primarily result in bone loading in this site. This new study shows that early walking is also associated with upper limb and spine bone strength in older age. Walking age can be regarded as a global marker of motor development, as greater locomotor ability in early life is also associated with performance of upper limb motor performance in later childhood.^4^ Therefore, it may be that walking age is also associated with upper limb physical activity, supported by associations between walking age and lean mass, although upper limb physical activity was not objectively assessed. Alternatively, stronger bones with greater cross‐sectional area could allow attachments of tendons of large muscles and thus expedite early independent walking. This interpretation is supported by the general enhancement of associations in model 2 (with adjustment for height), which reduces the allometric component that larger muscles are needed for longer bones, and further by the attenuation of associations when adjusted for lean mass. However, a previous study^3^ showed no association between bone strength at birth and walking age or between muscle and bone at birth. However, strong relationships were observed at 15 months, suggesting that these associations develop after birth rather than larger bones or muscles driving walking development.

That later walking age is also associated with lower bone strength at central and upper limb sites is important becasue fracture incidence is even greater in the upper than lower limbs and fractures of the central skeleton represent around a fifth of all fractures in older individuals;^23^ hence, maintenance of bone health throughout the skeleton is required to minimize fracture incidence. This study is the first to examine associations between walking age and detailed hip geometry. Advantages in bone strength in early‐walking men at both the predominately cortical shaft and predominately trabecular neck sites in this study appeared to result from greater bone size, cortical thickness, and BMD. In addition, advantages in femoral neck and shaft CSMI (indicating bone bending and torsional strength) were also evident from HSA analysis in women, whereas basic analysis did not identify any effects on BMC (indicating compressive bone strength).

### Potential explanations of findings

Because later walking age was associated with lower bone strength even after adjustment for early and adult life factors known to affect bone, it may be that there is a direct, persisting effect of walking age on bone strength. Associations between early life health and development indicators and later life health are well established, such as those observed between birth weight and bone strength in older age.^15^, ^24^ During growth, a lower limb bone growth “spurt” occurs at age 1 to 2 years, which cannot be explained by changes in body size.^25^ This peak (mirrored in the upper limbs slightly earlier at onset of crawling age^25^) occurs at about the time of walking onset, which is associated with large (∼40%) increases in BMC primarily through greater bone size.^3^ It appears that the ability to increase bone size via loading is impaired in adulthood^26^ but that advantages accrued during youth persist several decades after exercise cessation.^27^ Therefore it may be that early life advantages in bone size and strength in early walkers persist into adulthood. This appears to be supported by the pattern of bone differences between early and late walkers observed in this and a previous study,^4^ based on differences in bone size rather than density.

In this previous study, associations between later walking age and lower bone strength were partly explained by lower adolescent lean mass in later walkers.^4^ Greater lean mass is associated with higher levels of high‐impact physical activity,^28^ which in turn is associated with greater bone strength^5^, ^29^ in adolescents (particularly males). Given that associations between impaired motor development in early life and lower childhood physical activity are evident^30^ and persist into adolescence in males,^31^ it seems plausible that associations between walking age and bone strength could therefore act through altered motor development and physical activity. Unfortunately, no data on either childhood physical activity or lean mass in earlier life are available in the NSHD. However, adolescent sports ability (as an indicator of motor competence) was examined, but its inclusion resulted in only minor attenuation of associations between walking age and bone strength.

Given the lack of a sex difference in walking age, sex differences in associations cannot be explained by this variable per se but more likely by differential response to, for example, mediating effects of childhood loading. Early life motor development is associated with adolescent physical activity only in males,^31^ whereas effects of high‐impact physical activity on bone size and strength are greater in adolescent males than in females.^5^ This may relate to lower average levels of vigorous physical activity observed in adolescent females than in males, in addition to smaller interindividual variation.^31^ Hormonal effects could also underlie this sex difference.^32^

As with a previous study in adolescents,^4^ associations between walking age and bone strength were attenuated by adjustment for lean mass (and, to a much lesser extent, fat mass). However, given that models in both the previous and current studies were also adjusted for height, the mechanism of this mediation is unclear. Although physical activity is associated with lean mass in adolescents, relationships in older adults are less evident.^21^ There was little effect of adjustment for monitored or self‐reported physical activity data on associations between walking age and bone in the NSHD.

Because the influence of muscular action on bone is more important than the effects of external impacts^33^ and patterns of muscle and impact forces differ during movement,^34^, ^35^ it may be that muscle measures are acting as a more sensitive indicator of bone loading than accelerometry‐derived measures. This is supported by the far stronger relationships between lean mass and BMC, even when adjusted for height and fat mass,^36^ than accelerometer‐derived measures.^37^, ^38^ As with early life mediators, associations between walking age and bone strength could be explained by greater vigorous physical activity levels in males,^39^ in addition to greater bone mechanosensitivity.

### Significance and implications

The current results suggest that walking age may represent a risk factor for low bone strength in men. Height‐adjusted spine and total hip BMD were around 0.8 SD lower in men in the lowest sixth of walking age (16 months or older) compared with those in the highest sixth (10 months or younger). A 1 SD lower BMD is associated with approximately doubled fracture risk; hence, men walking at 16 months or later would be expected to have a ∼75% greater fracture risk than early walkers. Walking‐onset age is a modifiable factor, with even simple parent‐led exercise interventions producing earlier attainment of independent walking.^6^ Therefore, such interventions may represent an inexpensive route to improve lifelong bone health.

These results may have implications for a number of clinical groups such as preterm or small‐for‐gestational‐age children, whose prevalence has increased in recent decades.^40^, ^41^ These groups have delayed motor skill development accompanying low bone strength; hence, interventions aimed at improving early life motor competence may attenuate these deficits. For example, in children with Down syndrome, a home‐based, parent‐led treadmill training intervention improved onset of walking age by 4 months compared with controls.^7^ Given these large effects on walking age (which in turn are associated with higher bone strength in early childhood^3^), benefits to bone development could become evident through even small‐scale studies. This is supported by results of a recent trial in which walking practice led to improved bone mass in infants with myelomeningocele (a neural tube defect leading to pronounced delays in onset of independent walking^42^, ^43^). Results of the current and previous studies suggest that this would result from direct effects of earlier exposure to locomotory forces, in addition to mediation by the consequent improvements in motor competence, physical activity, and lean mass.^4^, ^28^, ^31^

### Strengths and limitations

Strengths of the study include unique assessment of associations between walking age and skeletal health at multiple fracture‐prone sites around six decades after walking onset. The availability of prospective data on a wide range of covariates across life and assessment of bone in all participants at a similar age are also strengths. However, given the observational nature of the study, causality cannot be attributed. Residual confounding by factors not included in analyses may explain these associations, but when other factors were considered (eg, birth order), they were not found to be important. Also, societal changes in areas such as health care, nutrition, and attitudes to physical activity may limit the ability to generalize these results to current or future generations. Age at onset of walking was recorded by questionnaire at age 2 years rather than at the time; however, parental recall of walking onset is shown to have excellent reliability up to 2 years after the event.^44^ Exclusion of missing data may have introduced bias; participants with data for all covariates included in the study were taller than participants with walking age and bone outcome data, although other exposures and outcomes were similar. Similar results were observed when analyses were rerun on maximum available samples, suggesting that effects on findings of these exclusions were limited.

### Conclusions

In conclusion, age at onset of independent walking appears to have a lifelong association with estimates of bone strength at multiple skeletal sites in men. In addition to associations with bone growth, these may be mediated by altered physical activity and lean mass. The lack of strong associations in women is another important finding from this study. These results suggest that early life interventions known to hasten walking onset may also have a positive influence on skeletal health across the population and in children. Given the challenges of length of follow‐up for verifying these findings in population‐level interventional trials, it would be prudent to first investigate these proposals in children prone to low bone strength and increased fracture risk.

## Disclosures

All authors state that they have no conflicts of interest.

## Supporting information

Supporting Table S1.
