# Supplementary material for: Later Age at Onset of Independent Walking Is Associated With Lower Bone Strength at Fracture‐Prone Sites in Older Men
Source: J Bone Miner Res. 2017 Mar 27;32(6):1209–17. doi: 10.1002/jbmr.3099 (PMC5466883; doi:10.1002/jbmr.3099)
Supplement: Supplementary file 1 — Supporting Table S1. [file JBMR-32-1209-s001.docx]

| Variable | | Men (n=588) | | | | Women (n=627) | | | |
| --- | --- | --- | --- | --- | --- | --- | --- | --- | --- |
|  |  | Beta | 95% CI | | *P* | Beta | 95% CI | | *P* |
| Birthweight (kg) | | -0.062 | -0.143 | 0.019 | 0.136 | -0.022 | -0.100 | 0.056 | 0.579 |
| Height (m) | | 0.083 | 0.002 | 0.164 | 0.044 | 0.076 | -0.002 | 0.154 | 0.056 |
| Weight (kg) | | -0.092 | -0.173 | -0.011 | 0.026 | -0.114 | -0.191 | -0.036 | 0.004 |
| Lean Mass (kg) | | -0.111 | -0.192 | -0.031 | 0.007 | -0.095 | -0.173 | -0.017 | 0.017 |
| Fat Mass (kg) | | -0.070 | -0.150 | 0.011 | 0.092 | -0.116 | -0.194 | -0.038 | 0.004 |
|  | | Mean | SD | | *P* | Mean | SD | | *P* |
| Father’s Occupational Class (age 4y) | I | 14.7 | 2.5 | | >0.001 | 14.3 | 2.4 | | <0.001 |
|  | II | 14.4 | 2.6 | |  | 14.1 | 2.4 | |  |
|  | IIINM | 13.9 | 1.9 | |  | 14.2 | 2.4 | |  |
|  | IIIM | 13.4 | 2.4 | |  | 12.9 | 2.1 | |  |
|  | IV | 12.9 | 2.2 | |  | 13.2 | 2.2 | |  |
|  | V | 13 | 2.2 | |  | 13.1 | 2.1 | |  |
| Sports Ability (age 13y) | Above average | 13.4 | 2.2 | | 0.045 | 13.2 | 2.1 | | 0.052 |
|  | Average | 13.7 | 2.4 | |  | 13.7 | 2.3 | |  |
|  | Below average | 14.1 | 2.5 | |  | 13.9 | 2.5 | |  |
| Own Occupational Class (age 53y) | I | 14.3 | 2.4 | | 0.019 | 13.6 | 2 | | 0.035 |
|  | II | 13.9 | 2.4 | |  | 13.7 | 2.2 | |  |
|  | IIINM | 13.6 | 2 | |  | 13.4 | 2.4 | |  |
|  | IIIM | 13.1 | 2.2 | |  | 13 | 2.6 | |  |
|  | IV | 13.5 | 2.7 | |  | 13.8 | 2.4 | |  |
|  | V | 13.7 | 1.8 | |  | 13.1 | 2.8 | |  |
| Leisure time physical activity (age 60-64y) | Inactive | 13.6 | 2.4 | | 0.701 | 13.6 | 2.4 | | 0.82 |
|  | Moderately active | 13.8 | 2.6 | |  | 13.7 | 2.1 | |  |
|  | Most active | 13.8 | 2.2 | |  | 13.5 | 2.2 | |  |

Supplementary Table 1. Associations between participant characteristics and age at onset of independent walking for men and women in the MRC National Survey of Health and Development. Associations are presented as standardised regression coefficients (representing the SD difference in walking age per 1SD increase in predictor) in the case of continuous variables, and as group mean age at onset of independent walking (months) and SD for categorical variables.

| Sex | Model | Total Hip | | | | | | | | Spine | | | | | | | |
| --- | --- | --- | --- | --- | --- | --- | --- | --- | --- | --- | --- | --- | --- | --- | --- | --- | --- |
|  |  | BMD | | | | BA | | | | BMD | | | | BA | | | |
|  |  | Beta | 95% CI | | P | Beta | 95% CI | | P | Beta | 95% CI | | P | Beta | 95% CI | | P |
| Men | 1 | -0.11 | -0.19 | -0.03 | 0.007 | -0.08 | -0.16 | 0.00 | 0.046 | -0.08 | -0.16 | 0.00 | 0.046 | -0.10 | -0.18 | -0.01 | 0.021 |
|  | 2 | -0.13 | -0.21 | -0.05 | 0.002 | -0.13 | -0.20 | -0.06 | <0.001 | -0.13 | -0.20 | -0.06 | <0.001 | -0.14 | -0.21 | -0.07 | <0.001 |
|  | 3 | -0.10 | -0.19 | -0.02 | 0.015 | -0.11 | -0.18 | -0.04 | 0.002 | -0.11 | -0.18 | -0.04 | 0.002 | -0.12 | -0.19 | -0.05 | <0.001 |
|  | 4 | -0.10 | -0.18 | -0.02 | 0.019 | -0.11 | -0.18 | -0.04 | 0.002 | -0.11 | -0.18 | -0.04 | 0.002 | -0.12 | -0.19 | -0.05 | 0.001 |
|  | 5 | -0.08 | -0.16 | 0.00 | 0.041 | -0.11 | -0.18 | -0.04 | 0.003 | -0.11 | -0.18 | -0.04 | 0.003 | -0.11 | -0.18 | -0.04 | 0.002 |
|  | 6 | -0.04 | -0.11 | 0.04 | 0.331 | -0.09 | -0.16 | -0.02 | 0.012 | -0.09 | -0.16 | -0.02 | 0.012 | -0.08 | -0.15 | -0.01 | 0.021 |
|  | 7 | -0.04 | -0.11 | 0.03 | 0.29 | -0.08 | -0.15 | -0.01 | 0.026 | -0.08 | -0.15 | -0.01 | 0.026 | -0.08 | -0.15 | -0.01 | 0.022 |
| Women | 1 | -0.03 | -0.11 | 0.05 | 0.42 | -0.03 | -0.11 | 0.05 | 0.41 | -0.03 | -0.11 | 0.05 | 0.48 | -0.04 | -0.11 | 0.04 | 0.35 |
|  | 2 | -0.04 | -0.12 | 0.04 | 0.30 | -0.04 | -0.12 | 0.04 | 0.31 | -0.05 | -0.13 | 0.02 | 0.16 | -0.08 | -0.14 | -0.01 | 0.03 |
|  | 3 | -0.03 | -0.11 | 0.05 | 0.44 | -0.03 | -0.11 | 0.05 | 0.45 | -0.05 | -0.13 | 0.03 | 0.19 | -0.08 | -0.15 | -0.01 | 0.03 |
|  | 4 | -0.03 | -0.11 | 0.05 | 0.53 | -0.03 | -0.11 | 0.06 | 0.54 | -0.05 | -0.13 | 0.03 | 0.20 | -0.08 | -0.15 | -0.01 | 0.02 |
|  | 5 | 0.00 | -0.09 | 0.09 | 0.97 | 0.00 | -0.09 | 0.09 | 0.97 | -0.01 | -0.09 | 0.06 | 0.70 | -0.06 | -0.13 | 0.01 | 0.09 |
|  | 6 | 0.01 | -0.07 | 0.09 | 0.78 | 0.01 | -0.07 | 0.09 | 0.79 | 0.00 | -0.08 | 0.07 | 0.96 | -0.05 | -0.11 | 0.02 | 0.17 |
|  | 7 | 0.01 | 0.01 | 0.02 | 0.74 | 0.01 | -0.06 | 0.09 | 0.74 | 0.00 | -0.07 | 0.07 | 0.98 | -0.05 | -0.11 | 0.02 | 0.18 |

Supplementary Table 2. Associations between age at onset of independent walking and total hip and spine DXA bone outcomes for men and women in the MRC National Survey of Health and Development. Associations are presented as standardised regression coefficients, representing the SD difference in each bone outcome per 1SD increase in age at walking. BMD – bone mineral density, BA – bone area.

Footnote: Model 1 predictors: Walking Age, Model 2: Model 1 + Height, Model 3: Model 2 + Birthweight + Father’s Occupational Class + Sports Ability, Model 4: Model 3 + Adult Occupational Class + Exercise, Model 5: Model 4 + Fat Mass, Model 6: Model 4 + Lean Mass, Model 7: Model 6 + Fat Mass + Lean Mass.

| Sex | Model | Femoral Shaft HSA | | | | | | | | Femoral Neck HSA | | | | | | | |
| --- | --- | --- | --- | --- | --- | --- | --- | --- | --- | --- | --- | --- | --- | --- | --- | --- | --- |
|  |  | BMD | | | | CT | | | | BMD | | | | CT | | | |
|  |  | Beta | 95% CI | | P | Beta | 95% CI | | P | Beta | 95% CI | | P | Beta | 95% CI | | P |
| Men | 1 | -0.12 | -0.20 | -0.04 | 0.005 | -0.09 | -0.17 | -0.01 | 0.022 | -0.10 | -0.18 | -0.01 | 0.022 | -0.09 | -0.17 | -0.01 | 0.026 |
|  | 2 | -0.13 | -0.21 | -0.05 | 0.002 | -0.10 | -0.18 | -0.02 | 0.015 | -0.11 | -0.19 | -0.03 | 0.009 | -0.10 | -0.18 | -0.02 | 0.012 |
|  | 3 | -0.10 | -0.19 | -0.02 | 0.014 | -0.08 | -0.17 | 0.00 | 0.049 | -0.10 | -0.18 | -0.02 | 0.018 | -0.10 | -0.18 | -0.01 | 0.022 |
|  | 4 | -0.10 | -0.18 | -0.02 | 0.016 | -0.08 | -0.17 | 0.00 | 0.052 | -0.10 | -0.18 | -0.01 | 0.023 | -0.09 | -0.18 | -0.01 | 0.029 |
|  | 5 | -0.08 | -0.16 | 0.00 | 0.037 | -0.07 | -0.14 | 0.01 | 0.108 | -0.09 | -0.17 | 0.00 | 0.04 | -0.08 | -0.17 | 0.00 | 0.049 |
|  | 6 | -0.03 | -0.10 | 0.04 | 0.394 | -0.02 | -0.10 | 0.05 | 0.585 | -0.05 | -0.13 | 0.03 | 0.19 | -0.05 | -0.13 | 0.03 | 0.215 |
|  | 7 | -0.04 | -0.11 | 0.04 | 0.341 | -0.02 | -0.10 | 0.05 | 0.526 | -0.06 | -0.14 | 0.03 | 0.184 | -0.05 | -0.13 | 0.03 | 0.207 |
| Women | 1 | -0.05 | -0.13 | 0.03 | 0.212 | -0.04 | -0.11 | 0.04 | 0.368 | -0.03 | -0.11 | 0.05 | 0.415 | -0.03 | -0.11 | 0.05 | 0.412 |
|  | 2 | -0.06 | -0.14 | 0.02 | 0.165 | -0.04 | -0.11 | 0.04 | 0.352 | -0.04 | -0.12 | 0.04 | 0.303 | -0.04 | -0.12 | 0.04 | 0.314 |
|  | 3 | -0.05 | -0.13 | 0.03 | 0.205 | -0.04 | -0.12 | 0.04 | 0.38 | -0.03 | -0.11 | 0.05 | 0.436 | -0.03 | -0.11 | 0.05 | 0.447 |
|  | 4 | -0.05 | -0.13 | 0.04 | 0.267 | -0.03 | -0.11 | 0.05 | 0.473 | -0.03 | -0.11 | 0.05 | 0.529 | -0.03 | -0.11 | 0.06 | 0.543 |
|  | 5 | -0.01 | -0.07 | 0.06 | 0.883 | 0.01 | -0.07 | 0.08 | 0.902 | 0.00 | -0.09 | 0.09 | 0.966 | 0.00 | -0.09 | 0.09 | 0.965 |
|  | 6 | -0.01 | -0.08 | 0.06 | 0.693 | 0.03 | -0.05 | 0.10 | 0.514 | 0.01 | -0.07 | 0.09 | 0.783 | 0.01 | -0.07 | 0.09 | 0.786 |
|  | 7 | -0.02 | -0.09 | 0.06 | 0.66 | 0.03 | -0.05 | 0.10 | 0.501 | 0.01 | 0.01 | 0.02 | 0.736 | 0.01 | -0.06 | 0.09 | 0.741 |

Supplementary Table 3. Associations between age at onset of independent walking and femoral neck and femoral shaft HSA bone outcomes for men and women in the MRC National Survey of Health and Development. Associations are presented as standardised regression coefficients, representing the SD difference in each bone outcome per 1SD increase in age at walking. BMD – bone mineral density, CT – cortical thickness.

Footnote: Model 1 predictors: Walking Age, Model 2: Model 1 + Height, Model 3: Model 2 + Birthweight + Father’s Occupational Class + Sports Ability, Model 4: Model 3 + Adult Occupational Class + Exercise, Model 5: Model 4 + Fat Mass, Model 6: Model 4 + Lean Mass, Model 7: Model 6 + Fat Mass + Lean Mass.

| Sex | Model | 4% Radius | | | | | | | |
| --- | --- | --- | --- | --- | --- | --- | --- | --- | --- |
|  |  | Total CSA | | | | Trabecular BMD | | | |
|  |  | Beta | 95% CI | | P | Beta | 95% CI | | P |
| Men | 1 | -0.11 | -0.19 | -0.03 | 0.008 | -0.10 | -0.18 | -0.01 | 0.021 |
|  | 2 | -0.14 | -0.22 | -0.07 | <0.001 | -0.14 | -0.21 | -0.07 | <0.001 |
|  | 3 | -0.13 | -0.21 | -0.05 | 0.002 | -0.12 | -0.19 | -0.05 | <0.001 |
|  | 4 | -0.12 | -0.20 | -0.04 | 0.002 | -0.12 | -0.19 | -0.05 | 0.001 |
|  | 5 | -0.11 | -0.19 | -0.03 | 0.005 | -0.11 | -0.18 | -0.04 | 0.002 |
|  | 6 | -0.07 | -0.14 | 0.00 | 0.056 | -0.08 | -0.15 | -0.01 | 0.021 |
|  | 7 | -0.07 | -0.15 | 0.00 | 0.054 | -0.08 | -0.15 | -0.01 | 0.022 |
| Women | 1 | 0.01 | -0.08 | 0.09 | 0.872 | -0.01 | -0.10 | 0.07 | 0.764 |
|  | 2 | -0.01 | -0.09 | 0.07 | 0.822 | -0.01 | -0.10 | 0.08 | 0.878 |
|  | 3 | -0.02 | -0.11 | 0.06 | 0.61 | 0.00 | -0.09 | 0.08 | 0.947 |
|  | 4 | -0.04 | -0.12 | 0.05 | 0.413 | 0.00 | -0.09 | 0.09 | 0.974 |
|  | 5 | -0.03 | -0.12 | 0.05 | 0.465 | 0.00 | -0.08 | 0.08 | 0.98 |
|  | 6 | -0.01 | -0.09 | 0.07 | 0.793 | -0.01 | -0.11 | 0.08 | 0.803 |
|  | 7 | -0.02 | -0.10 | 0.07 | 0.683 | 0.01 | -0.09 | 0.10 | 0.853 |

Supplementary Table 4. Associations between age at onset of independent walking and distal radius pQCT bone outcomes for men and women in the MRC National Survey of Health and Development. Associations are presented as standardised regression coefficients, representing the SD difference in each bone outcome per 1SD increase in age at walking. CSA – cross-sectional area, BMD – bone mineral density.

Footnote: Model 1 predictors: Walking Age, Model 2: Model 1 + Height, Model 3: Model 2 + Birthweight + Father’s Occupational Class + Sports Ability, Model 4: Model 3 + Adult Occupational Class + Exercise, Model 5: Model 4 + Fat Mass, Model 6: Model 4 + Lean Mass, Model 7: Model 6 + Fat Mass + Lean Mass.
